# Supplementary material for: Differences in species diversity, biomass, and soil properties of five types of alpine grasslands in the Northern Tibetan Plateau
Source: PLoS One. 2020 Feb 6;15(2):e0228277. doi: 10.1371/journal.pone.0228277 (PMC7004366; doi:10.1371/journal.pone.0228277)
Supplement: S1 Table — (DOC) [file pone.0228277.s001.doc]

**Supporting information**

**S1 Table. Community, species diversity and importance value in the five alpine grasslands**

| **Community types** | | | | | | | | | | | |
| --- | --- | --- | --- | --- | --- | --- | --- | --- | --- | --- | --- |
| **Main species** | **AM** | | | **AS** | | **AMS** | | **ADS** | **AD** | | |
|  | *Carex spp.*  (AM1) | *Poa*  *pratensis*  (AM2) | *Kobresia*  *humilis*  (AM3) | *Stipa capillata Linn.*  (AS1) | *Carex spp.*  (AS1) | *Stipa capillata Linn.* (AMS1) | *Artemisia argyi* (AMS2) | *Stipa capillata Linn.*  (ADS) | *Suaeda corniculate*  (AD1) | *Stipa capillata Linn.*  (AD1) | *Artemisia wellbyi*  (AD3) |
|  | Important value (IV) | | | | | | | | | | |
| ***Poa pratensis*** | 0.153 | 0.217 | 0.117 |  |  |  | 0.154 |  |  |  |  |
| ***Carex spp.*** | 0.257 | 0.153 | 0.158 | 0.27 | 0.238 | 0.216 | 0.215 | 0.257 |  |  |  |
| ***Kobresia humilis*** | 0.248 | 0.074 | 0.174 |  |  | 0.194 |  |  |  |  |  |
| ***Edelweiss*** | 0.134 | 0.152 | 0.168 | 0.186 | 0.174 |  |  |  |  |  |  |
| ***Oxytropis microphylla*** | 0.184 |  |  |  | 0.116 |  |  |  |  |  |  |
| ***Saussurea japonica*** |  | 0.137 | 0.123 |  |  |  |  |  |  |  |  |
| ***Potentilla chinensis*** |  | 0.138 | 0.129 | 0.228 |  |  |  | 0.153 |  |  |  |
| ***Stipa capillata Linn.*** |  |  |  | 0.277 | 0.19 | 0.309 | 0.163 | 0.476 |  | 0.391 | 0.095 |
| ***Artemisia argyi*** |  |  |  |  |  |  | 0.315 |  | 0.153 |  |  |
| ***Suaeda corniculate*** |  |  |  |  |  |  |  |  | 0.358 |  |  |
| ***Glaux maritima*** |  |  |  |  |  |  | 0.152 |  |  |  |  |
| ***Elymus dahuricus*** |  |  |  |  |  | 0.275 |  |  |  |  |  |
| ***Dandelion*** |  |  |  |  |  | 0.258 |  |  |  |  |  |
| ***Agrostis hugoniana Rendle*** |  |  |  |  |  | 0.197 | 0.201 |  |  |  |  |
| ***Oxytropis ochrocephala*** |  |  |  |  |  |  | 0.119 | 0.148 |  |  |  |
| ***Ptilotricum wageri*** |  |  |  |  |  |  |  | 0.154 |  |  |  |
| ***Artemisia macilenta*** |  |  |  |  |  |  |  | 0.122 | 0.136 |  |  |
| ***Artemisia wellbyi*** |  |  |  |  | 0.12 |  |  |  |  |  | 0.153 |
